# Thermal Characterization, Crystal Field Analysis and In-Band Pumped Laser Performance of Er Doped NaY(WO_4_)_2_ Disordered Laser Crystals

**DOI:** 10.1371/journal.pone.0059381

**Published:** 2013-03-21

**Authors:** María Dolores Serrano, Concepción Cascales, Xiumei Han, Carlos Zaldo, Andrzej Jezowski, Piotr Stachowiak, Nikolay Ter-Gabrielyan, Viktor Fromzel, Mark Dubinskii

**Affiliations:** 1 Department of Photonic Materials, Instituto de Ciencia de Materiales de Madrid, Consejo Superior de Investigaciones Científicas, Madrid, Spain; 2 Division of Low Temperature and Superconductivity, Institute of Low Temperature and Structure Research, Polish Academy of Sciences, Wroclaw, Poland; 3 Sensors and Electron Devices Directorate, United States Army Research Laboratory, Adelphi, Maryland, United States of America; University of Akron, United States of America

## Abstract

Undoped and Er-doped NaY(WO_4_)_2_ disordered single crystals have been grown by the Czochralski technique. The specific heat and thermal conductivity (*κ*) of these crystals have been characterized from *T* = 4 K to 700 K and 360 K, respectively. It is shown that *κ* exhibits anisotropy characteristic of single crystals as well as a *κ*(*T*) behavior observed in glasses, with a saturation mean free phonon path of 3.6 Å and 4.5 Å for propagation along *a* and *c* crystal axes, respectively. The relative energy positions and irreducible representations of Stark Er^3+^ levels up to ^4^G_7/2_ multiplet have been determined by the combination of experimental low (<10 K) temperature optical absorption and photoluminescence measurements and simulations with a single-electron Hamiltonian including both free-ion and crystal field interactions. Absorption, emission and gain cross sections of the ^4^I_13/2_↔^4^I_15/2_ laser related transition have been determined at 77 K. The ^4^I_13/2_ Er^3+^ lifetime (τ) was measured in the temperature range of 77–300 K, and was found to change from τ (77K) ≈ 4.5 ms to τ (300K) ≈ 3.5 ms. Laser operation is demonstrated at 77 K and 300 K by resonantly pumping the ^4^I_13/2_ multiplet at λ≈1500 nm with a broadband (FWHM≈20 nm) diode laser source perfectly matching the 77 K crystal ^4^I_15/2_ → ^4^I_13/2_ absorption profile. At 77 K as much as 5.5 W of output power were obtained in π-polarized configuration with a slope efficiency versus absorbed pump power of 57%, the free running laser wavelength in air was λ≈1611 nm with the laser output bandwidth of 3.5 nm. The laser emission was tunable over 30.7 nm, from 1590.7 nm to 1621.4 nm, for the same π-polarized configuration.

## Introduction

Lanthanide-doped disordered single crystals are receiving increasing attention in connection to their high potential as gain media in solid state lasers. The large optical transition bandwidth inherent to the coexistence of a distribution of Crystal Fields (CF) around the optical active ion has been found useful to better accommodate the emission bandwidth of diode lasers (DLs) presently used for optical pumping. This spectroscopic property is also greatly desired for mode-locked operation of ultrafast (fs) lasers. In comparison with glasses, single crystals usually have better thermo-optic and spectroscopic parameters, such as better thermal conductivity (*κ*) and higher emission cross sections (*σ*).

The disordered crystal families so far considered for this purpose include crystals with the hexagonal apatite structure Sr_2_Y_8_(SiO_4_)_6_O_2_ (melting point, m.p. 2173 K, *κ = *1.6//*a*-2.85//*c* Wm^−1^K^−1^) [1], tetragonal melilite SrLaGa_3_O_7_ (m.p. 2033 K, *κ = *1.95//*a*-1.7//*c* Wm^−1^K^−1^) [2], CaGaAlO_4_ (m.p. 1973 K, *κ = *6.9//*a*-6.3//*c* Wm^−1^K^−1^ for 2 at% Yb-doped) [3], oxoborates Ca_4_Gd(or Y)O(BO_3_)_3_ (m.p. 1753 K, *κ = *2.1 Wm^−1^K^−1^) [4], [5], [6], Ca_3_(NbGa)_2-x_Ga_3_O_12_ garnet (m.p. 1743 K, *κ = *4.7 Wm^−1^K^−1^ for 2 wt% Nd-doped) [7], [8], and tetragonal double tungstate NaY(WO_4_)_2_ (m.p. 1473 K, *κ = *1.062//*a*-1.166//*c* Wm^−1^K^−1^ for 5 at% Yb-doped) [9], [10], [11]. All these crystals melt congruently and can be grown by the technologically desired Czochralski method. The advantage of latter double tungstate is its significantly lower melting point which simplify the crystal growth (platinum instead iridium can be used as crucible material and air can be used as growth atmosphere). At room temperature most of these disordered crystals have relatively low thermal conductivity which has been reported to also decrease with doping, therefore active crystal cooling is important for stable high power laser operation.

Cryogenic cooling at 77 K is presently considered a viable means facilitating power scaling to a multi-kW class continuous wave (cw) laser operation [12]. The inconvenience of liquid nitrogen use is counterbalanced by performance improvements due to the increase of the peak absorption and emission cross sections of trivalent lanthanides (Ln^3+^) with temperature reduction. However, the spectral width of the Ln^3+^ absorption bands generally narrows with the increase of their peak absorption. This makes the use of disordered crystals more desirable in order to more fully utilize the optical power delivered by the pumping DLs. From this point of view crystals with medium CF strength are preferred over crystals with strong CF strength because at 77 K a compromise between the band linewidth and absorption intensity is expected to be found more easily.

To further reduce crystal heating during laser operation and relax cooling requirements resonantly (in-band) pumped solid state lasers are being developed. This pumping scheme minimizes the energy difference between the absorbed pump photons and those emitted by stimulated radiation, i.e., the crystals have low “quantum defect”. The most representative examples of this laser operation scheme are the ^2^F_7/2_ ↔ ^2^F_5/2_ operation of Yb^3+^ (λ≈1.06 µm) [13], and more recently ^5^I_8_ ↔ ^5^I_7_ operation of Ho^3+^ (λ≈2.07 µm) [14], ^3^F_3_ ↔ ^3^H_4_ operation of Pr^3+^ (λ≈1.65 µm) [15], and ^4^I_15/2_ ↔ ^4^I_13/2_ operation of Er^3+^ (λ≈1.60 µm) [16], [17], which are receiving increasing attention. The emission range around 1.60 µm is of particular interest for long range propagation in the atmosphere.

Therefore, laser operation of Ln^3+^-doped disordered crystals in-band pumped by DLs at 77 K is perceived as the most optimized approach to scaling the power of solid state lasers. Unfortunately, the growth and characterization of disordered crystals is still underdeveloped and their physical properties below room temperature are either unknown or not well established. In this work we studied the temperature evolution of the thermal parameters and spectroscopic characteristics of Er-doped NaY(WO_4_)_2_ single crystals below 300 K. We also demonstrated and characterized Er^3+^ laser operation in NaY(WO_4_)_2_ directly in-band pumped by InGaAsP/InP DLs at room and liquid nitrogen temperatures.

## Materials and Methods

### 1. Crystal Growth

Er-doped NaY(WO_4_)_2_ crystals were grown by the Czochralski method in air. The compound was previously synthesized by solid state reaction from Na_2_CO_3_ (99.5%), Y_2_O_3_ (99.9%), Er_2_O_3_ (99.99%) and WO_3_ (99.5%) starting materials. The starting compounds were annealed to eliminate moisture before weighting and later mixed in the appropriate composition. For synthesis, the mixture was first annealed to 1023 K for 18 h and cooled to room temperature, this product was ground and further annealed to 1123 K for 24 h. The phase of the resulting compound was monitored by powder X-ray diffraction. Afterwards, 1.5 wt% of Na_2_W_2_O_7_ was mixed with the synthesized Er-doped NaY(WO_4_)_2_ compound in order to decrease the melting temperature, to facilitate the crystal seeding and to compensate for possible evaporative loss of Na and W during the growth process. Overall, this provided a significant improvement in the optical crystal transparency.

**Figure 1:**
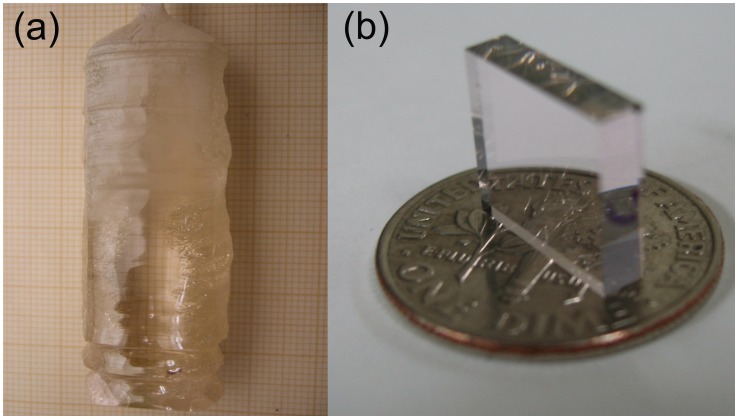
1 at% Er-doped NaY(WO_4_)_2_ crystal. (a) As grown boule. The small squares in the background scale are 1×1 mm^2^. (b) Polished sample used for laser experiments.

For crystal growth, this mixture was melted in a Pt crucible using a vertical resistive furnace. Undoped NaY(WO_4_)_2_ crystal oriented in the [100] direction was used as a seed. During the growth, the crucible temperature was related to the mass loss of the crucible to maintain constant crystal diameter. The seed rotation speed during the growth process was 10 r.p.m. and the crystal pulling rate was 1.6 mm/h. The grown crystal was cooled to room temperature at 10 K/h. Table 1 summarizes the composition of the grown crystals. Figure 1a shows an obtained crystal and Figure 1b shows a polished sample sliced from it and used for laser experiments.

**Table 1:** Erbium concentration, [Er], erbium segregation coefficient, *K*, and crystal lattice parameters of undoped and Er-doped NaY(WO_4_)_2_ crystals.

[Er]_MELT_	Region	[Er]_CRYS_	[Er]_CRYS_	*K*	*a*	*c*	*V*
(at%)		(at%)	(10^20^ cm^−3^)		(Å)	(Å)	(Å^3^)
0^a^					5.2014(4)	11.274(1)	305.01(5)
0.02	Top	0.030±0.02	0.0197±0.0013	1.5	5.1992(4)	11.272(1)	304.70(5)
1	Top	1.04	0.683	1.04	5.1981(5)	11.268(1)	304.46(6)
	Medium	1.04	0.683	1.04			
	Bottom	1.02	0.670	1.02			
3	Top	3.15	2.067	1.05	5.1964(8)	11.263(2)	304.13(9)
	Bottom	3.06	2.012	1.02			
15	Medium	14.7±0.7	9.68±0.46	0.98	5.1937(9)	11.261(2)	303.8(1)

a) REF 18.

**Figure 2:**
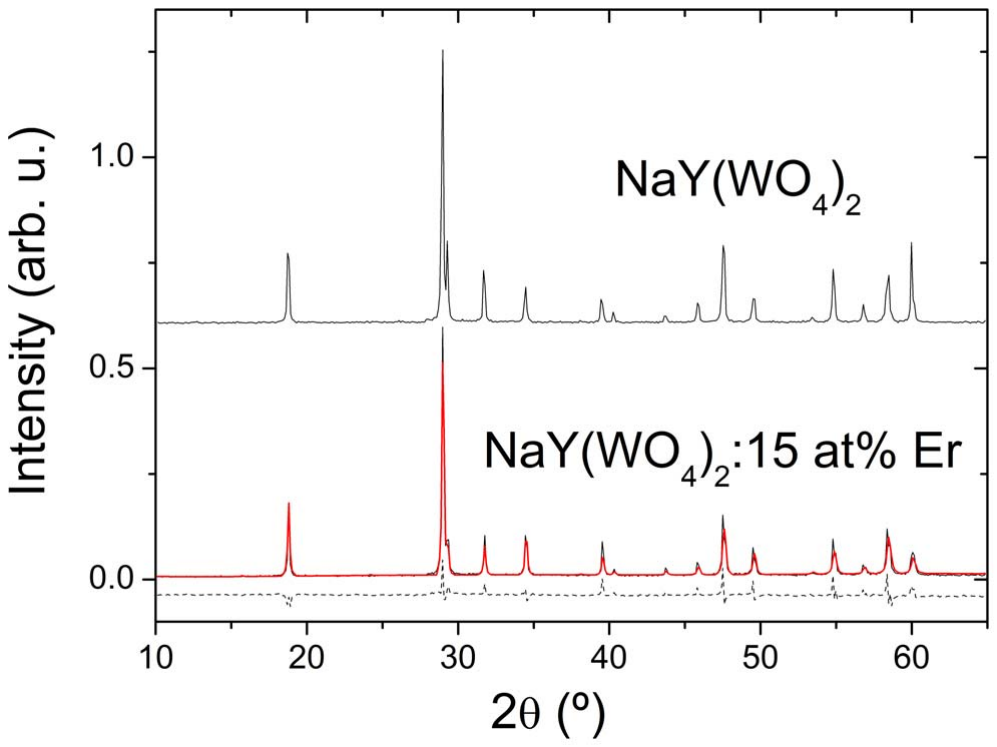
Powder X-ray diffraction scans of undoped and 15 at% Er-doped NaY(WO_4_)_2_ (black lines) crystals, Rietveld fit of the later results (red line) and its difference with the experimental data (dashed line).

The phase and crystalline structure of Er-doped single crystals were characterized by powder X-ray diffraction. For this purpose the single crystals were ground and diffraction scans were conducted at room temperature in a Bruker D8 Advance diffractometer. 2θ scans were acquired in the range 2θ = 10–65°, with a 2θ step of 0.05° and using an integration time of 1.5 s at each 2θ step. The results of these analyses were compared with those obtained for an undoped crystal [18] taken as reference. For clarity, Figure 2 only shows the comparison between undoped and 15 at% Er-doped crystals, the rest of Er compositions of Table 1 showed similar results. It is evident that Er-doped crystals are isostructural to the undoped reference. The structure of NaY(WO_4_)_2_ crystal was resolved by single crystal X-ray diffraction in a previous work [18]. It was shown that this crystal belongs to the tetragonal, no center-symmetric, space group with specific occupancy factors of 2*b* and 2*d* crystal sites simultaneously by the Na and Y (and Er) cations. Using this structural model, we carried out Rietveld refinements [19] to fit the experimental profile diffraction data. The difference between experimental and simulated data were small in all cases, see for instance Figure 2. The fits provided the crystal lattice parameters for each Er composition, see Table 1. As expected from the smaller ionic radii of eight-coordinated Er^3+^ (1.004 Å) than for Y^3+^ (1.019 Å), the lattice volume of the tetragonal cell decreases with increasing Er composition.

The Er concentration ([Er]) in the crystals with [Er] ≥ 1 at% was determined by X-ray fluorescence spectroscopy (XRFS) following procedures described previously [20]. The erbium concentration of the crystal with 0.02 at% Er in the melt was determined by comparison of its integrated 300 K ^4^F_9/2_ optical absorption with those of crystals with larger [Er]. The Er segregation coefficient (*K* = [Er ]_MELT_/[Er]_CRYSTAL_) is close to unity for all crystals with [Er ]_MELT_≥ 1 at% and it decreases slightly as the growth proceeds. This helps to preserve the melt composition and therefore to improve crystal quality and uniform distribution of erbium in the crystal. Hereafter, we will refer these crystals by their nominal Er composition (at%) in the melt.

### 2. Thermophysical Measurements

Specific heat at a constant pressure (*c_p_*) was measured below room temperature with a Quantum Design Physical Property Measurement system and above room temperature with a TA Instruments DSC Q-100 equipment. In the second case the isotherm operation mode was used, sapphire was taken as a reference and each reported result is the average of four measurements.

The evolution of the thermal conductivity with temperature *κ*(*T*) of the undoped, 1 at% Er-doped and 15 at% Er-doped NaY(WO_4_)_2_ crystals was measured by the steady-state longitudinal heat-flow method [21] in the *T* = 4–360 K range. Crystal samples in the form of rectangular bars with dimensions ≈3×3×15 mm^3^ were mounted in a liquid helium cryostat for the study of heat propagation along the long sample dimension, either *a* or *c* crystallographic axes. The temperature drop between the opposite sample faces did not exceed 0.2 K and extreme care was taken to eliminate parasitic heat flow between the sample and its environment. For this purpose a system of four thermal shields surrounding the sample was utilized and the temperature gradient along the innermost of them was maintained close to that one on the sample. Also, all necessary wires attached to the sample were thermally connected to this shield. To avoid an out of control heat loss due to convection, the measurements were performed under high vacuum (≈10^−7^ mbar) conditions.

### 3. Optical Spectroscopic Measurements

Optical absorption (OA) and photoluminescence (PL) measurements have been performed in the 7–300 K temperature range by using a closed-cycle He cryostat. OA was measured by a Varian spectrophotometer, model Cary 5E, whose light beam was filtered by using a Glan-Taylor calcite polarizer. For λ≈1.5 µm PL measurements the emission was dispersed in a SPEX 340E (*f* = 34 cm) spectrometer and detected with a Hamamatsu photomultiplier, model H9170-75, connected to a lock-in amplifier.

Further OA and PL measurements were made specifically at 77 K. In this case an Oxford flux cryostat operated with liquid nitrogen was used to assure temperature during OA experiments. The 77 K PL spectrum was obtained by illuminating a 0.02 at% Er-doped NaY(WO_4_)_2_ crystal with 970 nm DL emission and collecting the emission with a Yokogawa optical spectrum analyzer, model AQ6370. A polarization beam splitter was inserted between the sample and the collecting optics to separate π- and σ-polarized emissions.

According to the tetragonal character of the crystal the experimental spectra can be labeled as α (***E*** and ***B***⊥*c*-axis), σ (***E***//*a*-axis and ***B***//*c*-axis) or π (***E***//*c*-axis and ***B***//*a*-axis), where ***E*** and ***B*** are the electric and magnetic fields of the light, respectively.

### 4. Laser Measurements

Laser experiments were performed with a 1 at% Er-doped NaY(WO_4_)_2_ crystal with dimensions of 2.8×7×10 mm, see Figure 1b. The 7×10 mm faces were anti-reflection (AR) coated for the spectral range of 1480–1620 nm. For measurements at cryogenic temperatures, crystals were mounted on the copper cold finger inside a boil-off liquid nitrogen cryostat and cooled down to ∼79 K. For room temperature measurements, the crystals were placed on a water-cooled copper mount and were conductively cooled to 291 K.

The crystals were longitudinally pumped by a DL module which puts out a collimated beam delivering up to 17 W of CW π-polarized pump power with the output spectrum centered at 1501 nm. The non-narrowed output bandwidth of this InGaAsP/InP DL module used in our laser experiments was measured to be ∼20 nm FWHM. The module consists of 10 single emitters free-space-combined into a single polarized beam with the divergence of ∼7 mrad in both vertical and horizontal directions. Each InGaAsP/InP single emitter emits with the output spectral bandwidth of 10–12 nm FWHM, typical of this material, but multiple emitters for this module were not pre-selected to have the same peak wavelength position at the same temperature. So the integral spectral bandwidth of the module is ∼20 nm FWHM due to the dispersion in a single diode emitter peak positions. More details of the experimental laser methods are given later.

## Results and Discussion

### 1. Thermophysical Properties

**Figure 3:**
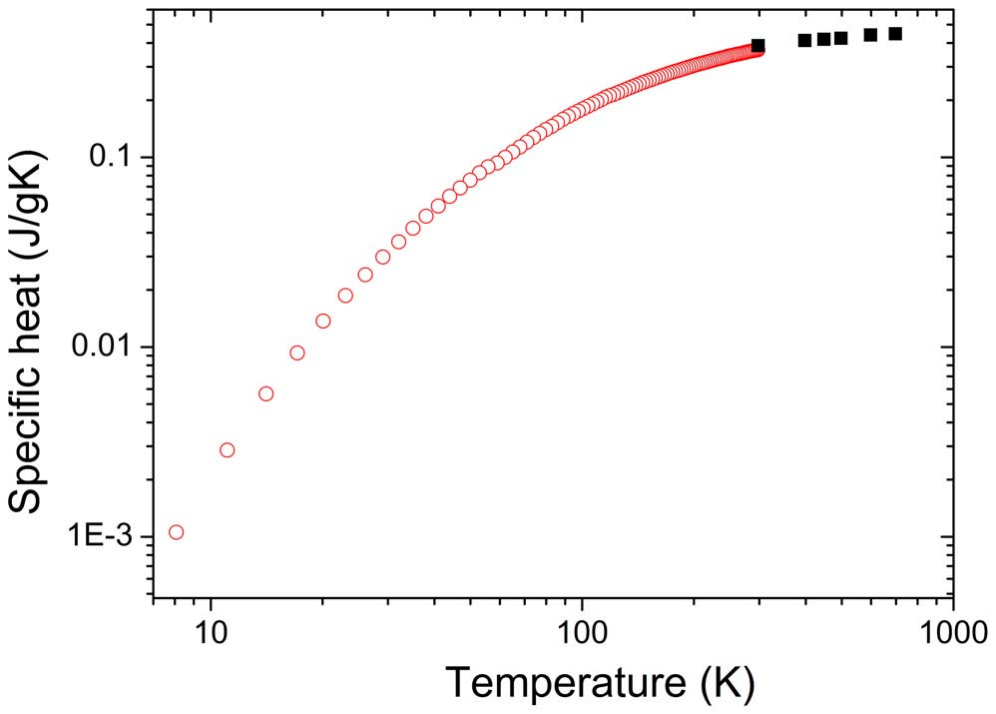
Temperature evolution of the specific heat (*c*_p_) of a 1 at% Er-doped NaY(WO_4_)_2_ crystal upon cooling from 700 K to 4 K. *c*_p_ for T >298 K, black solid squares; *c*_p_ for T<298 K, red open circles.

Figure 3 shows the results of the *c_p_* measurements obtained for the 1 at% Er-doped NaY(WO_4_)_2_ crystal. The specific heat shows the typical behavior of a dielectric crystal, both in terms of the dependence on temperature (*T*) and of its value.

**Figure 4:**
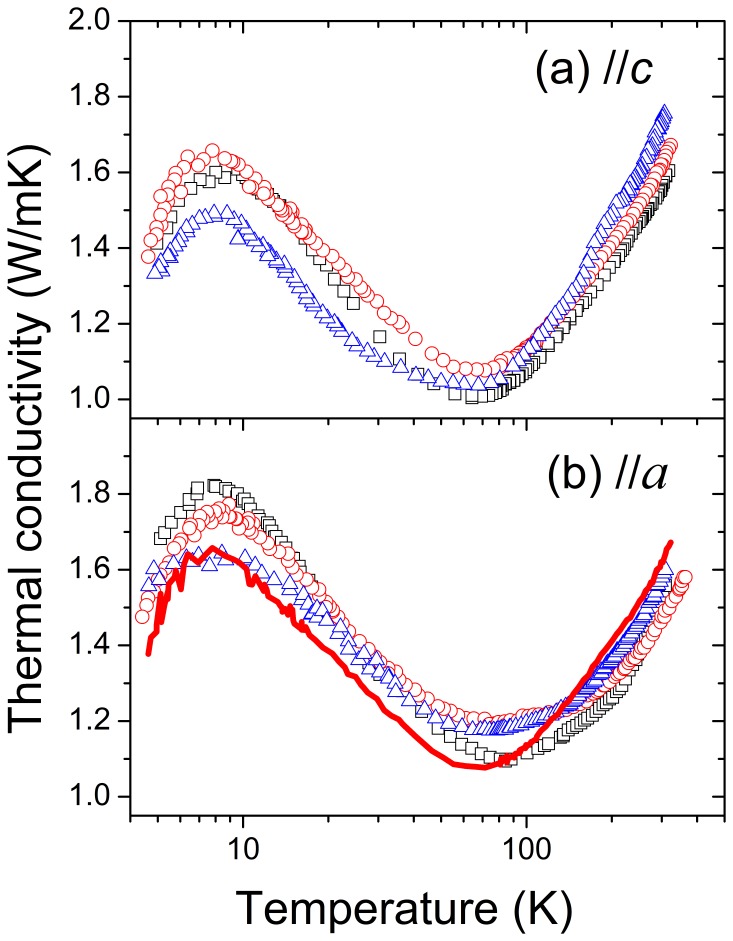
Evolution of the thermal conductivity of undoped (□), 1 at% Er− (○) and 15 at% Er− (Δ) doped NaY(WO_4_)_2_ crystals upon cooling from 360 K to 4 K. Heat propagation parallel to *c*-axis (a) and *a*-axis (b). The line in (b) shows for comparison the *κ*(*T*)*//c* of 1 at% Er-doped NaY(WO_4_)_2_ crystal.

Figure 4 shows the results of the *κ*(*T*) measurements. The *κ*(*T*) shape and value are very much different than those observed for a typical dielectric single crystal of relatively good quality. The *κ* change of the tested NaY(WO_4_)_2_ crystals does not exceed 100% over the entire investigated temperature range while typically *κ* changes by some orders of magnitude of the value. From low temperature *κ* increases with temperature and displays a maximum at *T* ≈ 8 K. Such a maximum is a typical feature of thermal conductivity of a crystal and it is due to the interplay between the increasing (with temperature) energy of phonons and increasing intensity of the three-phonon scatterings, also known as U-processes, in which the momentum of the created phonon is opposite to the sum of the momenta of the two interacting phonons, leading to a strong thermal resistivity [21]. For a dielectric crystal at temperatures well above the maximum the U-processes also dominate in the thermal conductivity and cause it to change as *T* ^−*η*^, where *η*≈1. However, in the NaY(WO_4_)_2_ crystals here investigated after the initial drop following the maximum, the thermal conductivity attains its minimum at around 70 K and then starts to increase again with temperature. This increase cannot be explained by typical lattice thermal transport mechanisms.

For further analysis of the experimental results we will utilize an expression originating from the kinetic theory of gases which allows us to write the thermal conductivity *κ* as
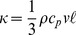
(1)where *ρ* = 6.616 Mg/m^3^ is the NaY(WO_4_)_2_ crystal density, *v* is the sound velocity (for NaY(WO_4_)_2_, *v*//*a* = 4700 m/s and *v*//*c* = 4190 m/s) [22] and *ℓ* is the phonon mean free path. From the expression given above one can find that with increasing temperature *ℓ* decreases down to a saturation constant value inducing a *κ* minimum near 70 K (for 1 at% Er-doped NaY(WO_4_)_2_, *κ//a* = 1.20 Wm^−1^K^−1^ and *κ//c* = 1.27 Wm^−1^K^−1^) and for higher temperatures the *κ*(*T*) evolution depends on *c_p_*(*T*). Taking into account the room temperature values of the thermal conductivity (for 1 at% Er-doped NaY(WO_4_)_2_, *κ//a = *1.47 Wm^−1^K^−1^ and *κ//c* = 1.62 Wm^−1^K^−1^) and *c_p_*(300 K) = 0.3868 Jg^−1^K^−1^, the phonon mean free path saturation values result *ℓ*//*a* = 3.6 Å and *ℓ*//*c* = 4.5 Å. The independence of the phonon mean free path of temperature in high temperature region is a glassy structure feature [23] (in crystals the phonon mean free path varies at these temperatures approximately as *T* ^−1^). Therefore, despite that from the macroscopic point of view the studied crystals exhibit the typical features of single crystals, like a marked anisotropy of the physical constants and well defined X-ray diffraction peaks, in terms of thermal properties NaY(WO_4_)_2_ is rather a glass-like structure than a crystal. At the lowest investigated temperatures, where one observes in glasses a plateau of the thermal conductivity of a small value of the coefficient *κ*≈ 0.1 Wm^−1^K^−1^ some “crystalline” characteristics of the investigated samples such as the little maximum discussed above appear. The dominant glassy character of the investigated crystals also explains the very little effect of Erbium doping on thermal conductivity. In view of small changes observed as Er concentration increases, the origin of the phonon scattering must be related to the presence of multiple local ionic arrangements due to the near to random distribution of Na and Y over the 2*d* and 2*b* sites of the NaY(WO_4_)_2_ crystalline structure. [18].

At room temperature the anisotropy of *κ* shown in Figure 4b (*κ//a*<*κ//c*) qualitatively agree with previous results obtained using the laser flash method for 5 at% Yb-doped NaY(WO_4_)_2_ [11] and Yb-doped NaGd(WO_4_)_2_ crystals [24] however the absolute *κ* values obtained by the heat-flow method are slightly larger. This anisotropy becomes smaller with sample temperature reduction and eventually its sign changes, i.e., below certain temperature *κ//a*>*κ//c*. This low temperature anisotropy behavior can be attributed to the distinct arrangement of the (Na/Y)O_8_ and WO_4_ polyhedra building the crystallographic structure of NaY(WO_4_)_2_. Along the [100] direction the more rigid WO_4_ polyhedra with short (≈1.8 Å) strong covalent W-O bonds alternate with (Na/Y)O_8_ polyhedra with considerably larger bond distance (≈2.4 Å), in contrast, the [001] direction is characterized by the presence of dimeric 2(Na/Y)O_8_ units sharing edges.

### 2. Er^3+^ Energy Levels

**Figure 5:**
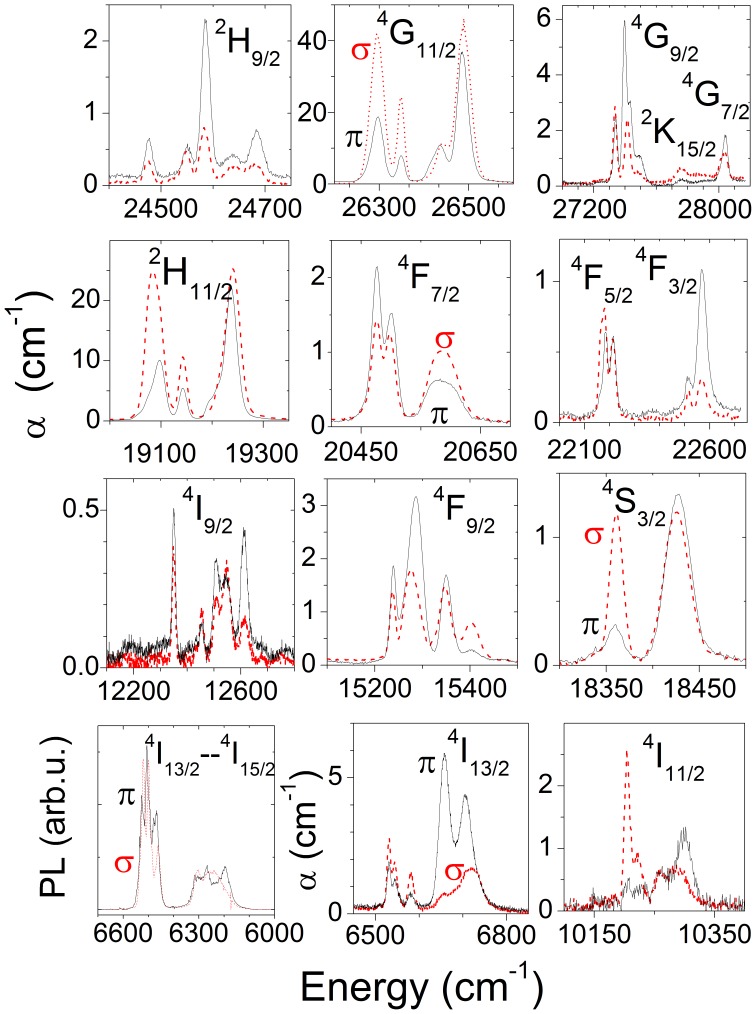
Low temperature (7 K) optical absorption coefficient (α) and photoluminescence (PL) of Er-doped NaY(WO_4_)_2_ crystal. σ-polarization, red dashed line; π-polarization, black solid line.

**Figure 6:**
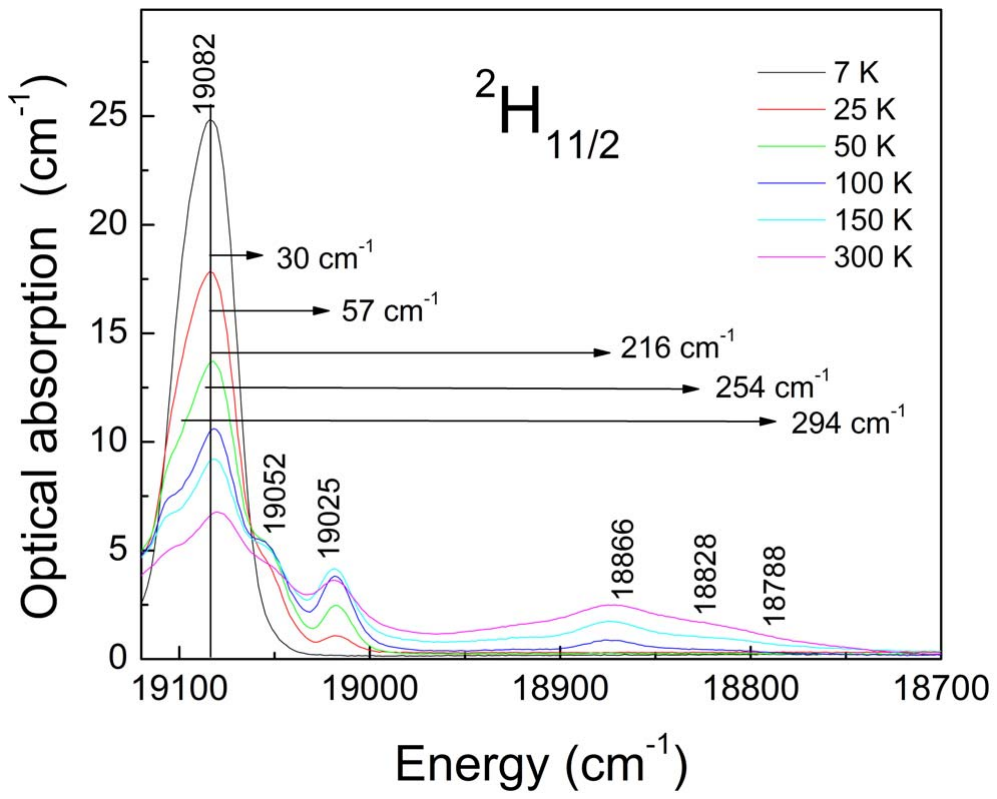
Thermal evolution of the ^4^I_15/2_→^2^H_11/2_ σ-polarized optical absorption of an Er-doped NaY(WO_4_)_2_ crystal.

The Er^3+^ Stark energy level sequence has been deduced from the analysis of 7–300 K OA and PL spectra. Stark levels of the ^2S+1^L_J_ excited multiplets were determined by their ground state ^4^I_15/2_→^2S+1^L_J_ OA. The sublevels of the ^4^I_15/2_ multiplet were determined from the ^4^I_13/2_ →^4^I_15/2_ λ ≈ 1.5 µm PL measured in the 0.02 at% Er-doped NaY(WO_4_)_2_ crystal under excitation at λ = 520 nm (^4^I_15/2_→^2^H_11/2_), in this crystal PL re-absorption is expected to be minimized. Figure 5 shows the experimental 7 K OA coefficient (*α*) and PL results of Er-doped NaY(WO_4_)_2_ crystal and Table 2 summarizes the relative energies of the observed Stark levels. Moreover, the 7–300 K evolution of the ^2^H_11/2_ OA, shown Figure 6, was used to confirm and complement the 7 K PL measurements.

**Table 2:** Observed (E_o_) and calculated (E_c_) energy levels (cm^−1^) of Er^3+^ in NaY(WO_4_)_2_ crystal, in an average S_4_ symmetry.

^2S+1^L_J_	∥^a^	IR^b^	E_o_	E_c_		^2S+1^L_J_	∥^a^	IR^b^	E_o_	E_c_
^4^I_15/2_	σ	Γ_5,6_	0	−4		^4^F_7/2_	σ π	Γ_7,8_	20476	20483
		Γ_7,8_	–	10			σ	Γ_5,6_	20501	20495
		Γ_5,6_	30	36			σ	Γ_5,6_	20575	20575
		Γ_7,8_	57	52			σ π	Γ_7,8_	20592	20596
		Γ_7,8_	216	214						
		Γ_7,8_	254	246		^4^F_5/2_	σ	Γ_5,6_	22167	22167^c^
		Γ_5,6_	294	291		(^2^D1_5/2_)	σ	Γ_5,6_	22180	22181^c^
		Γ_5,6_	–	322			σ π	Γ_7,8_	22214	22215^c^
^4^I_13/2_	σ π	Γ_7,8_	6531	6540		^4^F_3/2_	σ	Γ_5,6_	22516	22519^c^
	σ	Γ_5,6_	6543	6540		(^2^D1_3/2_)	σ π	Γ_7,8_	22569	22562^c^
	σ	Γ_5,6_	6581	6579						
	σ π	Γ_7,8_	6658	6656		^2^G1_9/2_	σ π	Γ_7,8_	24475	24465^c^
	σ	Γ_5,6_	6681	6681		(^4^F_9/2_,^2^H2_9/2_)	σ	Γ_5,6_	24548	24561
	σ π	Γ_7,8_	6705	6708			σ π	Γ_7,8_	24583	24598^c^
	σ	Γ_5,6_	6721	6728			σ	Γ_5,6_	24642	24613
							σ π	Γ_7,8_	24684	24692^c^
^4^I_11/2_	σ π	Γ_7,8_	10204	10197^c^						
(^2^H2_11/2_)	σ	Γ_5,6_	10205	10209		^4^G_11/2_	–	Γ_5,6_	–	26299
	σ	Γ_5,6_	10221	10253^ c^		(^2^H2_11/2_)	σ π	Γ_7,8_	26295	26304^c^
	σ π	Γ_7,8_	10260	10274^ c^			–	Γ_7,8_	26349	26353
	σ	Γ_5,6_	10280	10281			σ	Γ_5,6_	26440	26438^c^
	σ π	Γ_7,8_	10300	10294^ c^			σ π	Γ_7,8_	26485	26482^c^
							σ	Γ_5,6_	26489	26494
^4^I_9/2_	σ π	Γ_7,8_	12350	12336						
(^2^H2_9/2_)	σ	Γ_5,6_	12453	12445		^4^G_9/2_	–	Γ_5,6_	27335	27333
	σ	Γ_5,6_	12508	12508		(^2^H2_9/2_)	σ π	Γ_7,8_	27340	27337^c^
	σ π	Γ_7,8_	12545	12514^c^			–	Γ_7,8_	27396	27391
	σ π	Γ_7,8_	12612	12612			σ	Γ_5,6_	27413	27408
							σ π	Γ_7,8_	27429	27422
^4^F_9/2_	σ π	Γ_7,8_	15239	15243^c^						
(^4^I_9/2_)	σ	Γ_5,6_	15275	15280		^2^K_15/2_	–	Γ_5,6_	27476	27481
	σ π	Γ_7,8_	15287	15286			σ π	Γ_7,8_	27492	27481
	σ π	Γ_7,8_	15350	15349			σ	Γ_5,6_	27758	27755
	σ	Γ_5,6_	15403	15404			–	Γ_7,8_	27762	27755
							–	Γ_7,8_	–	27794
^4^S_3/2_	σ	Γ_5,6_	18361	18365^c^			σ	Γ_5,6_	27844	27856
(^2^P_3/2_)	σ π	Γ_7,8_	18427	18424^c^			σ π	Γ_7, 8_	–	27891
							–	Γ_5,6_	–	27892
^2^H2_11/2_	σ	Γ_5,6_	19083	19083^c^						
(^4^G_11/2_)	σ π	Γ_7,8_	19097	19108^c^		^4^G_7/2_	σ	Γ_5,6_	28015	28027^c^
	σ π	Γ_7,8_	19142	19150^c^		(^2^G1_7/2_)	σ π	Γ_7,8_	28039	28045^c^
	σ	Γ_5,6_	–	19202^c^			σ	Γ_5,6_	–	28044
	σ π	Γ_7,8_	–	19212^c^			σ π	Γ_7,8_	–	28045
	σ	Γ_5,6_	19241	19223^c^						

a) OA observed polarization character (∥).

b) Irreducible representation (IR).

c) Levels with heavily mixed wavefunctions.

The crystal structure of NaY(WO_4_)_2_ contains two sites, 2*b* and 2*d,* with S_4_ point symmetry that are shared by Na^+^, Y^3+^ and Er^3+^ [18]. Despite some specific short-range Na^+^ and Y^3+^ distributions which can even suppose a site symmetry lower than S_4_ around Er^3+^ centres, the observed Er^3+^ optical transitions shown in Figure 5 still retain the well defined S_4_ polarization character with regard to the principal axes of the matrix. Each Stark level ^2S+1^L_J_(ń) (which is double degenerated, i.e. Kramers doublets) can be labeled with an irreducible representation (IR), either Γ_5,6_ or Γ_7,8_. The number of bands (each one with an IR) expected for a given Er^3+4^I_15/2_(0)→^2S+1^L_J_(ń) transition is summarized in Table 3. The polarization state of the light required to be absorbed for a given transition is described in Table 4. Only electric dipole (ED) transitions are considered since the α and σ spectra were found equivalent. σ-spectrum shows all transitions independently of the IR of the initial and final states. Contrary, π-spectra only show transitions between levels with different IRs. The empirical analysis of these results is not free of uncertainty: a) For some few multiplets, for instance ^4^I_15/2_, ^2^H_11/2_ or ^4^G_11/2_, the number of observed bands is lower than that of the corresponding excited Stark levels. b) The IR of the ground ^4^I_15/2_(0) Stark level can not be directly concluded since no excited multiplet with J = 1/2 exists.

**Table 3:** Irreducible representations (IR) observed for S_4_ symmetry.

J	IR
1/2	Γ_7,8_
3/2	Γ_5,6_ + Γ_7,8_
5/2	2 Γ_5,6_ + Γ_7,8_
7/2	2 Γ_5,6_ + 2 Γ_7,8_
9/2	2 Γ_5,6_ + 3 Γ_7,8_
11/2	3 Γ_5,6_ + 3Γ_7,8_
13/2	4 Γ_5,6_ + 3 Γ_7,8_
15/2	4 Γ_5,6_ + 4 Γ_7,8_
17/2	4 Γ_5,6_ + 5 Γ_7,8_

**Table 4:** Selection rules for induced electric dipole ED and magnetic dipole MD transitions for the S_4_ symmetry.

IR	ED		MD	
	Γ_5,6_	Γ_7,8_	Γ_5,6_	Γ_7,8_
Γ_5,6_	α,σ	α,σ,π	α,σ, π	α, π
Γ_7,8_	α,σ, π	α,σ	α, π	α,σ, π

In order to rationalize the Er^3+^ spectroscopy, the energies of the Stark levels from ^4^I_15/2_ up to ^4^G_7/2_ were simulated using a one-electron CF model considering an average S_4_ potential described previously [25]. This potential simultaneously considers both free-ion (FI) and CF effects by using the entire basis set of 4f^11^ wavefunctions. Er^3+^ observed energy levels (E_o_) were distributed in two submatrices according to their experimentally assigned IR, which were diagonalized separately in the fitting process. The total Hamiltonian includes 26 parameters, but among them some FI parameters were held constant through the fit, while others were constrained to vary within determined limits, and the number of CF parameters imposed by the symmetry is quite reduced, only five real 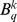 and one complex. Thus, a reasonable calculation of all interactions is assured with the sufficient number of experimentally determined Er^3+^ Stark levels.

The final simulation reproduces very adequately the experimental level sequence, with an overall agreement of σ = 10.7 cm^−1^, and in no case individual large discrepancies between E_o_ and calculated (E_c_) energy levels were found. This uncertainty is small considering the precision of the experimental data inherent to the large linewidth. The calculated energies are summarized in Table 2, and Table 5 reports the adjusted FI and CF parameters. The confidence in obtained phenomenological parameters and the physical meaning of the fit is also supported by the very similar results of previous calculation performed for the same 4f^11^ configuration in other isostructural MT(XO_4_)_2_ (M = Li or Na, T = Bi, X = Mo or W) hosts [25], [26] or for the closer 4f^13^ Yb^3+^ configuration in the same host [27].

**Table 5:** FI and CF parameters (cm^−1^) for Er^3+^ in NaY(WO_4_)_2_ crystal.

Parameter	Value
E^0^	35092 (1)
E^1^	6527.0 (7)
E^2^	32.81 (2)
E^3^	676.45 (8)
α	21.18 (3)
β	[−434]
γ	[1790]
ζ	2363.1 (9)
M^0, a)^	5.8 (5)
P^2, b)^	850 (15)
T^2^	[400]
T^3^	4 (2)
T^4^	450 (3)
T^6^	−327(15)
T^7^	483 (12)
T^8^	[299]
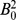	385 (19)
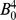	−673 (36)
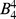	*±*862 (22)
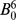	−147 (34)
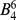	*±*517 (22)
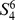	*±*189 (40)
l	66
d_m_	9.3
σ ^c)^	10.7
Residue	5654.6

Values in parentheses refer to estimate standard deviations in the indicated parameter. Values in square brackets were not allowed to vary in the parameter fitting.

a M^0^, M^2^, M^4^ were constrained by the ratios M^2^ = 0.56 M^0^, M^4^ = 0.32 M^0^.

b P^2^, P^4^, P^6^ were constrained by the ratios P^4^ = 0.75 P^2^, P^6^ = 0.50 P^2^.

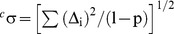
, Δi = Eo−Ec, l number of levels, p number of parameters.

The partition functions (*Z*) of the ^4^I_15/2_ and ^4^I_13/2_ multiplets involved in the 1.5 µm emission can be obtained from the level energies summarized in Table 2, as
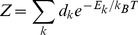
(2)where d_k_ = 2 because of the double degeneracy of the Kramers doublets, *E_k_* is the energy of a given Stark level with respect to the minimum energy of its multiplet, *k_B_* is the Boltzmann constant and *T* is the temperature. The Z values obtained for the ^4^I_15/2_ and ^4^I_13/2_ multiplets are 9.38 and 9.20, respectively.

### 3. Spectroscopic Properties Related to Resonantly Pumped ∼1.6 µm Laser

**Figure 7:**
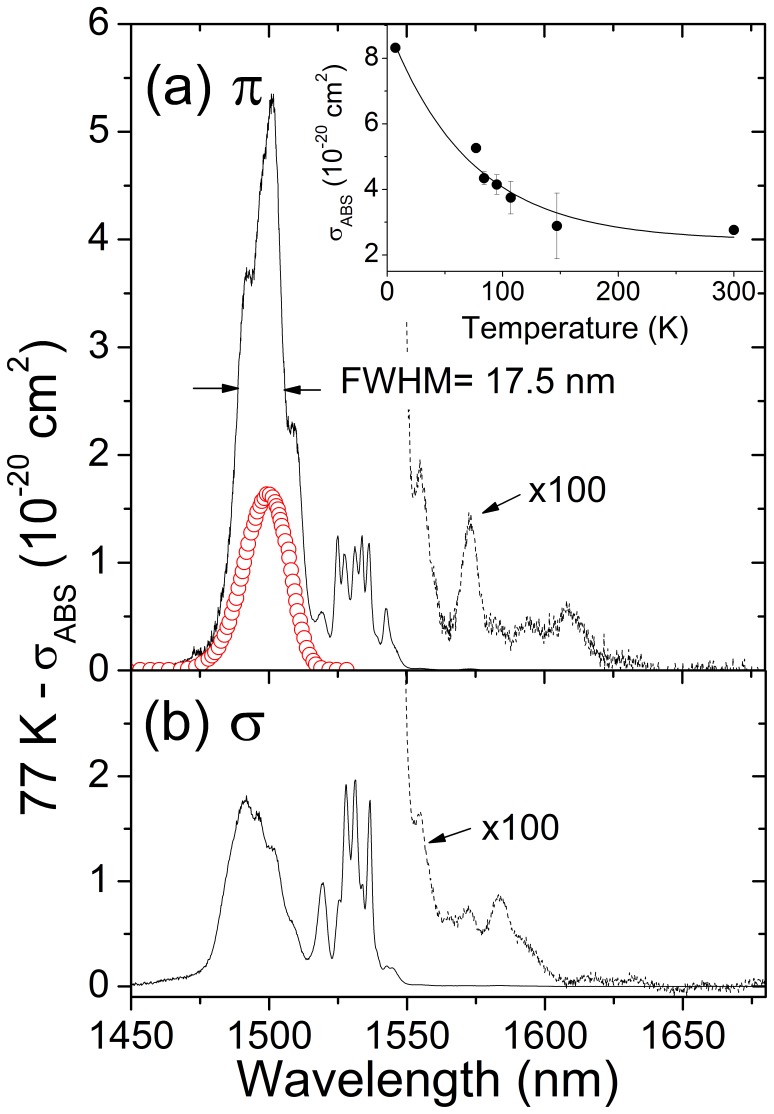
77 K ^4^I_13/2_ absorption cross section of Er-doped NaY(WO_4_)_2_ single crystal (lines) for π (a) and σ (b) polarizations. The non-narrowed emission of the InGaAsP/InP diode laser is shown for comparison (red points). The inset shows the thermal evolution of the absorption cross section at λ = 1501 nm for π-polarized light, the points are the experimental results and the line is the fit to an exponential function.

*T = *77 K is a convenient cryogenic cooling temperature because of the wide availability of liquid nitrogen that can be distilled from air. The purpose of this section is to evaluate the spectroscopic parameters of Er^3+^ in NaY(WO_4_)_2_ at this temperature. Figure 7 shows the 77 K ^4^I_13/2_ absorption cross section, *σ*_ABS_ = *α*/[Er], for σ- and π-polarization. The largest absorption, *σ*_ABS_ = 5.3±0.2×10^−20^ cm^2^, is obtained at λ = 1501 nm for π-polarization with a full width at half maximum (FWHM) for the convolution of the several overlapping peaks of 17.5 nm.

The reduction of the sample temperature from room temperature (300 K) to liquid nitrogen temperature (77 K) induces significant increase of the peak absorption cross section of the ^4^I_15/2_ → ^4^I_13/2_ transition, and a nearly structureless broad absorption band is observed at 1501 nm, see Figure 7a. The absorption at 77 K is composed by overlapping of several peaks and it shows a nearly perfect matching with the emission spectrum of our spectrally non-narrowed InGaAsP/InP DL module with the spectral bandwidth of FWHM ≈ 20 nm, used for pumping the Er^3+^:NaY(WO_4_)_2_ laser.

Larger peak cross sections can be obtained by further sample cooling up to the liquid He temperature, but this develops two well resolved bands and a central minimum with an absorption efficiency half of that corresponding to the nearby maxima, therefore the emission of the DL cannot be absorbed so efficiently for a given sample thickness. At the λ = 1501 nm fixed wavelength the sample absorption follows an exponential function related to the Boltzmann distribution of the electronic population of the ^4^I_15/2_ multiplet, see inset of Figure 7a.

The emission cross-section on the ^4^I_13/2_ → ^4^I_15/2_ transitions were obtained by stitching up the results of the reciprocity [28] (1470–1551.8 nm)
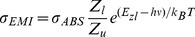
(3)(*E_zl_* is the energy of the 0→0′ transition) and Fuchtbauer-Landenburg [29] (1551.8–1640 nm) methods using the measured PL and lifetime (see below) of the ^4^I_13/2_ manifold:
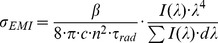
(4)where τrad is the radiative lifetime of the 4I_13/2_ state of Er^3+^, n is the refractive index [30] of the crystal, I(λ) is the fluorescence intensity in arbitrary units, λ is the average emission wavelength, c is the speed of light and β is the branching ratio corresponding to the transition 4I_13/2_ →4I_15/2_ (β = 1).

**Figure 8:**
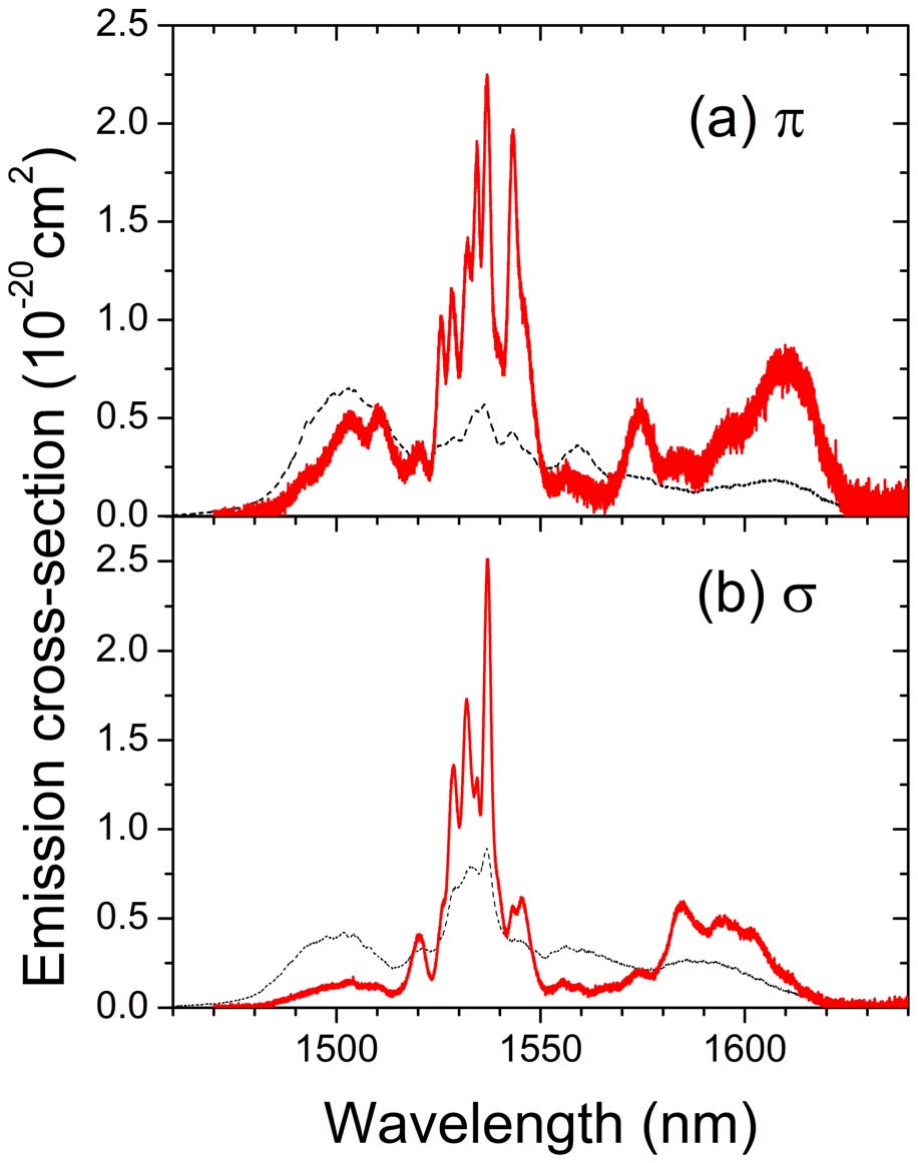
Polarization resolved emission cross sections of the ^4^I_13/2_ → ^4^I_15/2_ transition of Er^3+^ in NaY(WO_4_)_2_ single crystal measured at 77 K (red continuous line) and 300 K (black dashed line): (a) π-polarized spectra, (b) σ-polarized spectra.

Figure 8 shows the polarization resolved ^4^I_13/2_ → ^4^I_15/2_ emission cross sections of Er-doped NaY(WO_4_)_2_ crystal in the wavelength range of 1470–1640 nm at 77 K and 300 K. The peak emission cross-section of Er-doped NaY(WO_4_)_2_ crystal at 77 K in the 1560–1630 mm wavelength region for π-polarization (0.8×10^−20^ cm^2^) is slightly higher than that for σ-polarized ones (∼ 0.65×10^−20^ cm^2^) and it is shifted to long-wavelength region compared with σ-polarized emission spectrum.

**Figure 9:**
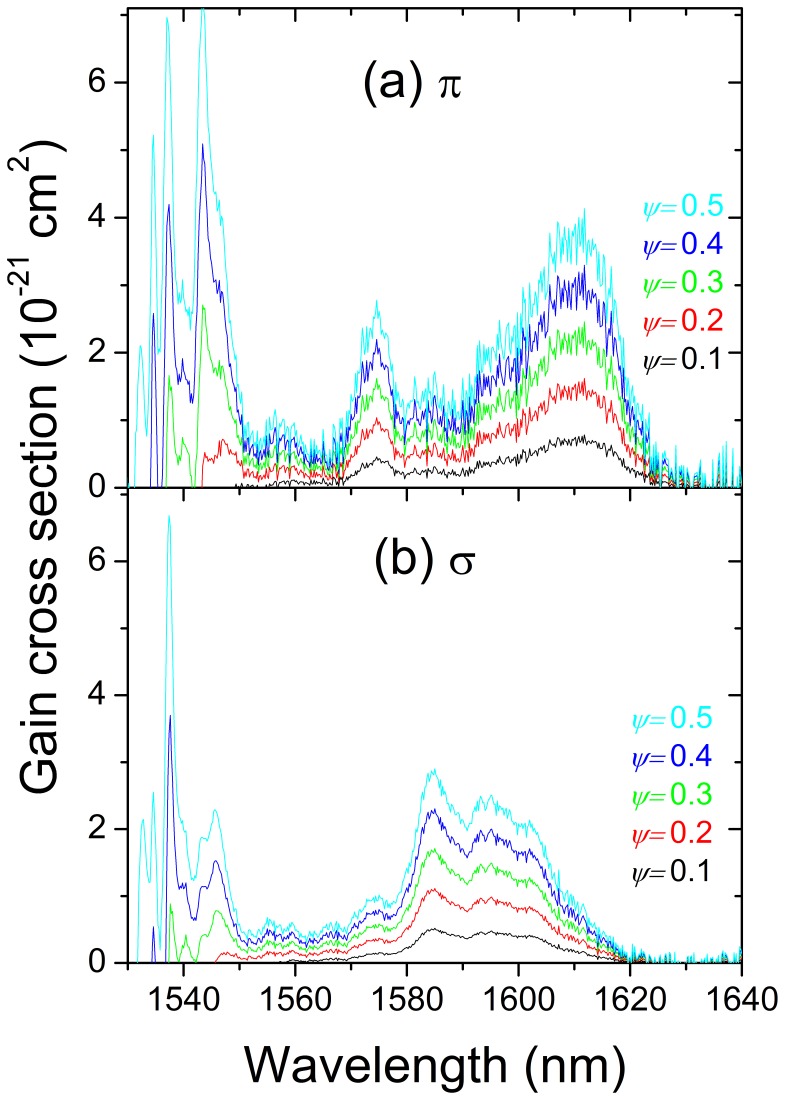
Gain cross sections of Er-doped NaY(WO_4_)_2_ crystals at 77 K for different inversion ratios, *Ψ*: (a) π-polarization, (b) σ-polarization.

The gain cross section *σ*_GAIN_ = *Ψσ*_EMI_-(1−*Ψ*)*σ*_ABS_, *Ψ* being the ratio between Er^3+^ ions in the excited state versus the total Er^3+^ concentration, provides first information about the lasing capability of resonantly pumped laser systems. Figure 9 shows the polarization resolved *σ*_GAIN_ results obtained from data of Figures 7 and 8. Two lasing regions can be distinguished, the first one around 1540 nm is characterized by narrow bands and it requires high inversion ratios, *Ψ*>0.3. The second one, extending from 1560 nm to 1625 nm for both π-polarized and σ-polarized configurations, occurs even for low *Ψ* values and it is characterized by broad bands, indicating of significant laser tunability potential (and femtosecond laser operation) in this wavelength range.

**Figure 10:**
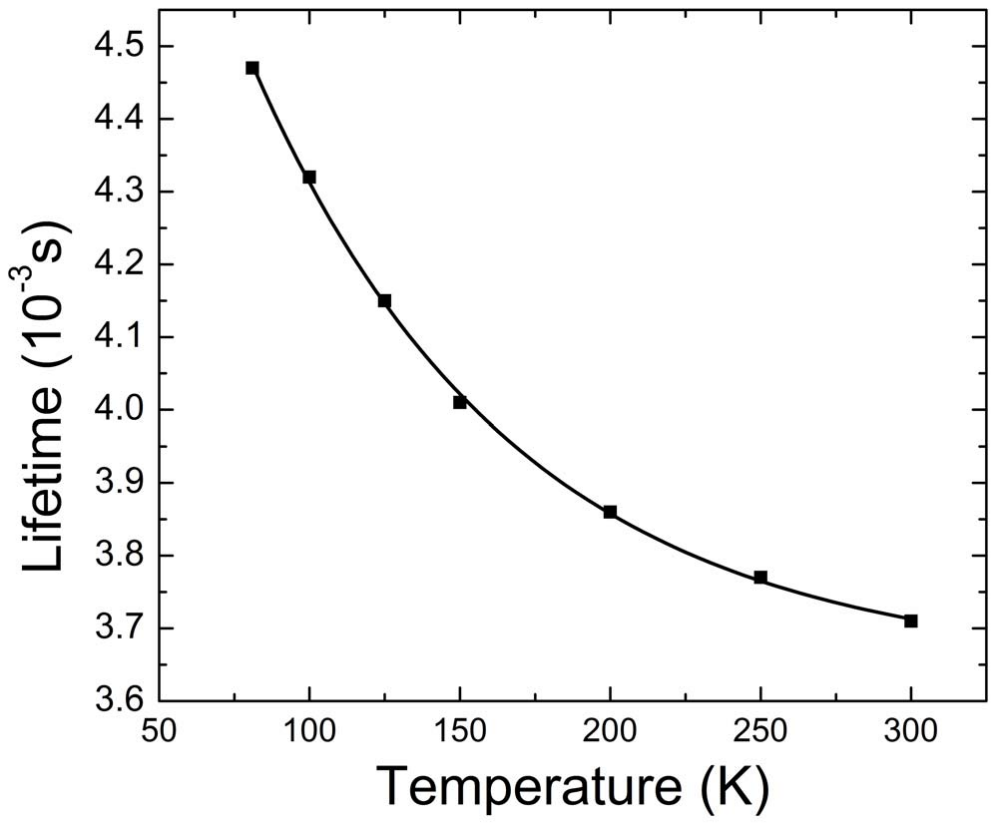
The lifetime of the ^4^I_13/2_ manifold versus temperature for the pulverized 0.02 at% Er-doped NaY(WO_4_)_2_ crystal.

Figure 10 shows the lifetime dependence on temperature of the upper laser level (^4^I_13/2_) of the 0.02 at% Er-doped NaY(WO_4_)_2_ crystal. The lifetime measurements were done on a pulverized sample with low concentration of Er^3+^ ions (≈1.9×10^18^ cm^−3^) in order to avoid effects of radiation trapping and fluorescence reabsorption on measurement results.

### 4. Resonantly Pumped Laser Experiments

**Figure 11:**
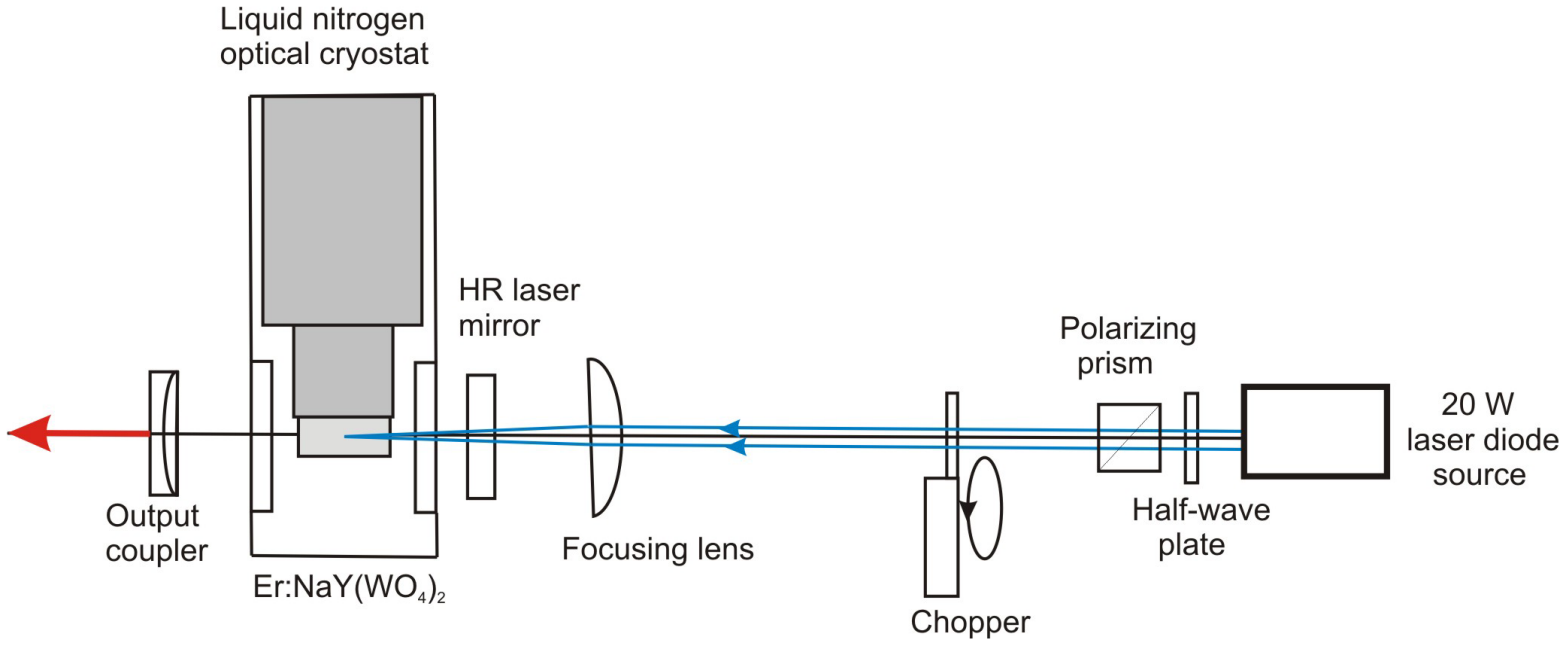
Simplified optical layout of the cryogenically cooled Er-doped NaY(WO_4_)_2_ laser.

Figure 11 shows a schematic representation of experimental setup and optical cavity used for laser characterizations.

**Figure 12:**
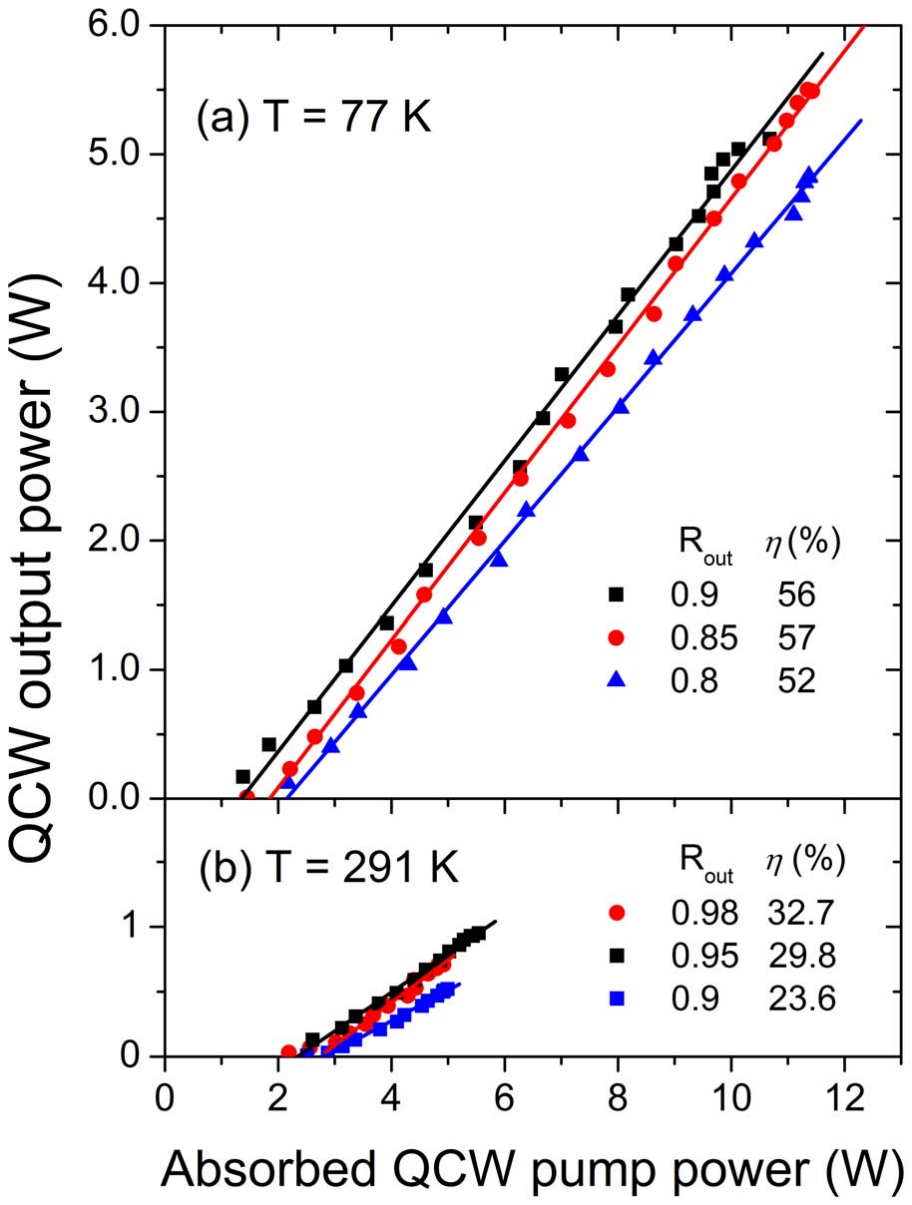
Laser output power vs. absorbed pump power (both quasi-CW) for the resonantly pumped 1 at% Er-doped NaY(WO_4_)_2_ laser (points). Quasi-CW regime: pulse duration 10 ms, pulse repetition frequency 10 Hz. (a) Cooled to 77 K. (b) Cooled at room temperature. The legend shows the reflectance of the used output coupler, R_out_, and the slope efficiency, *η*, obtained from the linear fits (lines).

The quasi-CW performance of the 1 at% Er-doped NaY(WO_4_)_2_ laser resonantly pumped into the 1501 nm absorption band (corresponding to ^4^I_15/2_(0) → ^4^I_13/2_(4) transition, see Table 2) at 77 K is shown in Figure 12a for three different OC reflections. Without a wavelength selective element in the cavity the laser operated in π-polarization. The Er-doped NaY(WO_4_)_2_ laser power in this case is presented versus absorbed pump power since the fraction of the absorbed pump power was observed to significantly vary with pump power due to saturation effects (averaging from 0.83 to ∼ 0.7 depending on the output coupler reflectivity and the pump density). .

The best output of 5.5 W and a slope efficiency *η = *57% versus absorbed pump power have been achieved with the cavity length of 12 cm, RoC of the OC ∼250 mm and OC reflectance of 85%. The TEM_00_ mode size in this case was about 500 µm along the entire crystal length and provided the best fit with the pumped volume. The measured laser beam divergence was about 3.3 mrad, which is close to a calculated value of the TEM_00_ mode divergence for the used laser cavity configuration. The π-polarized output spectrum of the Er-doped NaY(WO_4_)_2_ laser (taken with an optical spectrum analyzer) was centered at ∼ 1611 nm (air) and had a bandwidth of ∼3.5 nm, thus the laser operates with the quantum defect of ∼7%. The measured composite passive loss in the cavity, including cryostat windows, was found to be ∼4% for a single-pass at 1610 nm and was mainly introduced by the AR coatings on the crystal.

The evidence of the laser efficiency improvement upon cryo-cooling comes from comparison of Figures 12a and 12b. The output of the laser in Fig. 12b is also represented versus the absorbed pump power (both Q-CW with the same duty factor of 0.1). The best laser performance was achieved with the cavity length of ∼8 cm and RoC of the concave OC of 100 mm. The pump beam was focused into the crystal by the lens with the focal length of 75 mm. The diameter of the TEM_00_ laser mode along the crystal in this resonator was ∼ 340 µm, i.e. slightly smaller than the diameter of the pumped volume. The maximum obtained laser slope efficiency at room temperature with respect to absorbed pump power was 32.7% with the output coupler reflectivity of 98%. This value of the slope efficiency at room temperature is nearly twice higher than that reported recently by Huang, et al., for Er:Yb:Ce:NaY(WO_4_)_2_ laser [31]. The fraction of the pump power absorbed in the crystal with respect to incident pump power at room temperature was measured to be 0.65–0.55, depending on the output coupler reflectivity and the pump density. It is noticeably lower than that at 77 K, however, not as low as one could expect from comparison of the absorption spectra at cryogenic and room temperatures, see Figure 7. We believe that the reason for this is much smaller influence of the saturation effects on laser performance at room temperature. Maximum laser output power obtained at room temperature was ∼1 W. The laser output spectrum at room temperature was centered at 1609.6 nm (air) with the bandwidth of ∼4 nm.

To assess the tunability potential of the Er-doped NaY(WO_4_)_2_ laser, we performed tuning experiments with cryogenically cooled (77 K) Er-doped NaY(WO_4_)_2_ laser by inserting a three-stage birefringent tuner in the cavity between the laser crystal and the output coupler. The wavelength tuning was measured separately for two laser polarizations. For the σ-polarized laser output, the tuning range was measured to be ∼22 nm, from 1588 nm to 1610.2 nm, with the maximum at λ = 1595 nm. For the π-polarization the tuning curve was wider, around 30.7 nm, from 1590.7 nm to 1621.4 nm, with the maximum at 1610 nm. Both tuning curves correspond well with the calculated gain cross section profiles of Er-doped NaY(WO_4_)_2_ crystal for σ- and π-polarizations, see Figure 9. The measured tuning range was obviously limited by the coating of the cavity optics preventing lasing in the λ≈1540 nm region. This wide wavelength tuning range of Er-doped NaY(WO_4_)_2_ laser is very beneficial and will potentially support generation of ultra-short laser pulses with pulse durations down to ∼100 fs.

### Conclusions

The thermal conductivity of disordered NaY(WO_4_)_2_ crystal has a behavior resembling that observed in glasses, i.e., after the mean free path of phonons saturates to a constant value the recovery of the thermal conductivity is related to the increase of the specific heat with temperature. Despite this fact, the thermal conductivity retains the characteristic anisotropy of single crystals and is little affected by doping with Er ions, which is indicative of the principal phonon scattering processes association with the near to random distribution of Na and Y(or Er) in two possible crystal lattice sites, 2*b* and 2*d*. Therefore this behavior is expected to be also found in lasers doped with other lanthanides. Although it is well known that dopants (Er and other laser lanthanides) reduce the thermal conductivity of the laser crystals, the present results suggest that a further reduction of the thermal conductivity could be expected in disordered crystals based on the coexistence of several sites for the laser dopants. The extension of similar thermal measurements to disordered laser crystals mentioned in the Introduction section seems very necessary to quantify the relative magnitude of both possible thermal conductivity reduction mechanisms.

From low temperature (<10 K) polarized optical absorption and photoluminescence measurements assisted by the assessment with an energy simulation including the erbium free ion and crystal field interactions the relative energy sequence of the Er^3+^ Stark levels and their irreducible representations have been determined up to the ^4^G_7/2_ multiplet. Further, the 77 K absorption, emission and gain cross sections have been determined. It was shown that at 77 K the main absorption at λ = 1501 nm (π-polarized) perfectly fits the spectral distribution of the non-narrowed diode laser module used for resonant optical pumping of Er-doped NaY(WO_4_)_2_ laser. The lifetime of the upper ^4^I_13/2_ multiplet of Er^3+^ ions is ≈4.5 ms at 77 K and gets reduced to ≈3.7 ms at room temperature.

Erbium lasing may occur in narrow bands around 1550 nm or continuously between 1560 nm and 1625 nm. It was shown that by cooling the crystal to 77 K the maximum output power can be increased by a factor of five and the slope efficiency (versus absorbed power) by a factor of two with respect to laser operation at room temperature. The best resonantly pumped laser efficiency was obtained at 77 K by using a near to constant TEM_00_ cavity mode and pump mode size of about 500 µm of diameter along the entire crystal length. In this case the maximum achieved output power was 5.5 W with a slope efficiency (versus absorbed power) of *η = *57%. The laser output was π-polarized and centered at λ≈1611 nm with a FWHM of ≈3.5 nm. Laser tuning of over 30.7 nm (from 1590.7 nm to 1621.4 nm) has been demonstrated at 77 K for π-polarization. The tuning range for σ-polarization was slightly narrower and limited by the coatings of the cavity optics.
